# Evolution of the R2 Retrotransposon Ribozyme and Its Self-Cleavage Site

**DOI:** 10.1371/journal.pone.0066441

**Published:** 2013-09-16

**Authors:** Danna G. Eickbush, William D. Burke, Thomas H. Eickbush

**Affiliations:** Department of Biology, University of Rochester, Rochester, New York, United States of America; Louisiana State University, United States of America

## Abstract

R2 is a non-long terminal repeat retrotransposon that inserts site-specifically in the tandem 28S rRNA genes of many animals. Previously, R2 RNA from various species of Drosophila was shown to self-cleave from the 28S rRNA/R2 co-transcript by a hepatitis D virus (HDV)-like ribozyme encoded at its 5' end. RNA cleavage was at the precise 5' junction of the element with the 28S gene. Here we report that RNAs encompassing the 5' ends of R2 elements from throughout its species range fold into HDV-like ribozymes. *In vitro* assays of RNA self-cleavage conducted in many R2 lineages confirmed activity. For many R2s, RNA self-cleavage was not at the 5' end of the element but at 28S rRNA sequences up to 36 nucleotides upstream of the junction. The location of cleavage correlated well with the types of endogenous R2 5' junctions from different species. R2 5' junctions were uniform for most R2s in which RNA cleavage was upstream in the rRNA sequences. The 28S sequences remaining on the first DNA strand synthesized during retrotransposition are postulated to anneal to the target site and uniformly prime second strand DNA synthesis. In species where RNA cleavage occurred at the R2 5' end, the 5' junctions were variable. This junction variation is postulated to result from the priming of second strand DNA synthesis by chance microhomologies between the target site and the first DNA strand. Finally, features of R2 ribozyme evolution, especially changes in cleavage site and convergence on the same active site sequences, are discussed.

## Introduction

The structure and mechanism of self-cleaving RNAs and their catalytic role in various biological processes have been extensively reviewed [1]. The Hepatitis delta virus (HDV) encodes two such ribozymes with similar structures that function in its replication [2,3]. The crystal structures of these ribozymes revealed five paired helices folded into a double pseudoknot that constrains the structure and fashions the active site [4,5]. *In vitro* selection experiments based on the HDV ribozyme [6,7,8] and the subsequent discovery of the HDV-like CPEB3 ribozyme [9] revealed few invariant positions in this enzyme. Bioinformatics searches using the constraints on the HDV ribozyme secondary structure and sequence revealed similar ribozymes present in a wide range of organisms with many found upstream of reverse transcriptase sequences [10]. The proximity of many of these putative ribozymes to reverse transcriptase suggested self-cleavage had a role in the lifecycle of retrotransposable elements.

Non-long terminal repeat (non-LTR) retrotransposons comprise one of the two major families of transposable elements whose movement requires reverse transcriptase. How these elements are transcribed remains poorly understood. Some elements have been shown to encode a promoter, which initiates RNA synthesis at the precise 5' end of each element, while other elements are suggested to rely on co-transcription with cellular genes. Strong support for the latter was obtained when catalytically active HDV-like ribozymes were found in many non-LTR elements [11,12,13].

**Figure 1:**
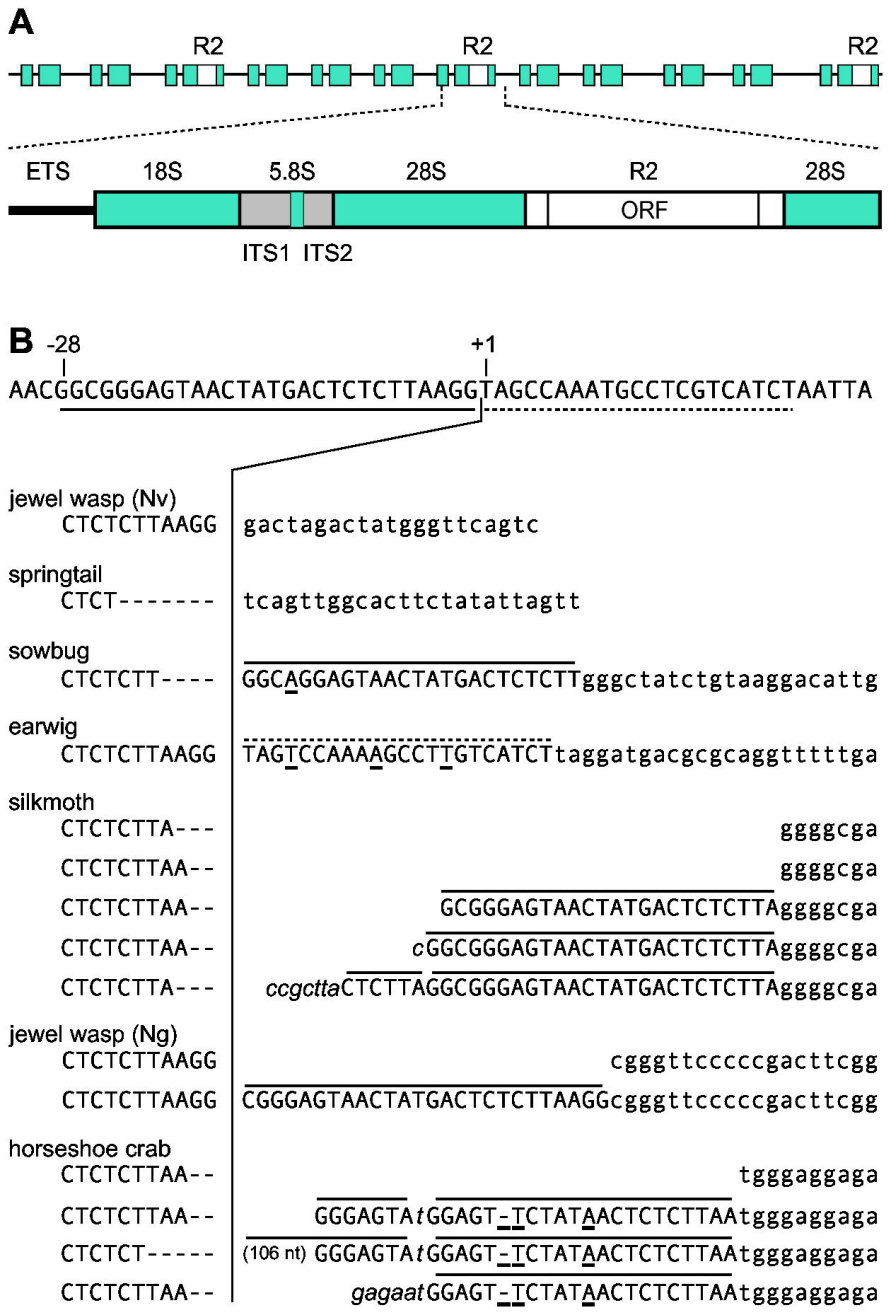
R2 junction variation. (A) A schematic representation of the tandem units in the ribosomal DNA locus with some units containing an R2 insertion. The rRNA transcription unit with external transcribed spacer (ETS), 18S, 5.8S and 28S genes (blue boxes), internal transcribed spacers (ITS, gray boxes), and R2 element (white box) is diagrammed. The single open reading frame (ORF) of R2 is indicated. (B) 28S gene sequence flanking the R2 insertion site with the conserved site of the initial nick indicated as position +1. The 5' junction sequences of the R2 elements obtained from seven arthropods are shown below the 28S sequence. Nucleotides to the left of the vertical line are upstream 28S gene sequences (uppercase). Nucleotides to the right of the vertical line are either R2 (lowercase), have identity to the 28S gene (uppercase, differences underlined), or non-templated additions (italics). One horseshoe crab junction contained an additional 106 nt that are not shown. Overlined sequences correspond to duplications of the 28S gene from upstream (solid) or downstream (dashed) of the target site.

R2 elements are among the best characterized non-LTR retrotransposable elements. These elements insert site-specifically in the tandemly arrayed 28S rRNA genes (Figure 1A) of most arthropods and appear to have been vertically inherited since the origin of this phylum [14,15]. Because the 28S rRNA target site is well conserved across eukaryotes, it has been straightforward to identify R2 in additional diverse animals [16], [Stage DE, unpublished data]. The mechanism of R2 propagation has been elucidated in depth and has provided a general model for non-LTR retrotransposition [17,18,19]. The encoded protein of the R2 element from *Bombyx mori* has been shown to bind both the 3' untranslated region (UTR) of its RNA and the DNA target site of the 28S gene, and through symmetric cleavage/synthesis reactions, termed target primed reverse transcription (TPRT), generate new copies. An analysis of R2s in multiple Drosophila species revealed HDV-like ribozymes at the 5' end of each element responsible for cleaving the 5' end of the R2 sequences from the 28S/R2 co-transcript. Surprisingly, the Drosophila R2 ribozymes not only shared the same secondary structure but also many of the nucleotides in the catalytic region with the HDV ribozyme [11,12].

The R2 ribozyme also plays an integral role in the generation of non-autonomous elements that are propagated by the R2 machinery. These parasites have been found in several Drosophila species and have been called Short Internally Deleted Elements (SIDEs) [20]. The SIDEs encode only the R2 ribozyme to process their 5' end from the 28S co-transcript and sequences with identity to the 3' UTR of R2 which are recognized by the R2 machinery in order to initiate reverse transcription.

The R2s of Drosophila, like many non-LTR retrotransposons, are inherently imprecise in integrating their 5' end to the DNA target site. The 5' junction sequences of full-length R2 elements from 14 species of Drosophila revealed that less than 40% had the canonical 5' junction [21]. The remaining junctions contained variable length deletions of the 28S gene and/or the addition of non-templated nucleotides upstream of the R2 sequences. This variation is not propagated through retrotransposition cycles but is generated at each insertion because R2 self-cleavage of the template RNA eliminates all upstream 28S gene sequences as well as any non-templated sequences [11].

A previous survey of arthropod R2s revealed that all elements encoded comparable proteins and had uniform 3' junctions located at the identical position within the 28S rRNA gene [22]. Thus, the first steps of R2 integration, attachment at the 3' end, are similar in all species. However, in many species the R2 5' junctions showed sequence variation unlike that observed in Drosophila suggesting different mechanisms of 5' attachment. It was of interest, therefore, to determine whether the R2 elements in these species also encode 5' self-cleaving ribozymes and whether differences in the activity of these ribozymes contributed to the difference in the 5' junctions.

Here we report the presence of active HDV-like ribozymes in the R2s of divergent animals. Unlike the ribozyme that is entirely encoded in the R2 elements of Drosophila, the ribozyme associated with R2 processing in many animals contains sequences from the 28S gene upstream of the R2 target site. This ribozyme configuration results in self-cleavage upstream in the 28S gene sequences rather than at the 5' end of the R2 element. We propose a model that explains how the upstream rRNA sequences give rise to more precise 5' end attachment. We also note aspects of the evolution of the R2 ribozyme in animal phyla and detail the flexibility in structure permitted for the R2 ribozyme.

## Materials and Methods

### PCR amplification of 5' junctions

Genomic DNA from most species was previously isolated [22,23]. *Tribolium* *castaneum* (red flour beetle) specimens were obtained from Carolina Biological Supply. Individual beetles were crushed in 100 µl of buffer (10 mM Tris HCl, pH 8.2; 1 mM EDTA; 25 mM NaCl; 20 µg proteinase K), incubated for 30 minutes, and then boiled for 2 minutes. The R2 5'/ 28S junctions from all species were generated by PCR amplification by pairing the T7/28S(-95) primer [5'- TAATACGACTCACTATAGGGCACAATGTGATTTCTGCCCAGT-3'] with species-specific R2 primers at various positions within the element. The *Dong* 5' junction was generated by PCR amplification by pairing the T7/TAA/Dong primer [5'-TAATACGACTCACTATAGGGTAATAATAATAATAATAATAAGCTCCCTAAAATCCTACC-3'] with *Dong* specific primers. Sequences of the species-specific element primers can be found in Supporting Information, Text S1. PCR fragments, with the exception of the *T* *. castaneum* R2 and *Dong* 5' ends, were cloned into the pCR2.1-TOPO cloning vector (Invitrogen) and sequenced (Macrogen).

### DNA templates for T7 co-transcription/cleavage reactions

DNA templates for RNA transcription were generated by PCR amplification with unincorporated primers and nucleotides removed using a PCR Purification Kit (BioBasics). Assays were preformed as previously described [11]. Approximately 0.1 µg of PCR template was incubated in transcription buffer with 20 units of T7 RNA Polymerase (Invitrogen) and trace amounts of [α-^32^P] UTP for one hour at 42°C. Reactions were then placed on ice and 4 volumes of 95% formamide, 10 mM EDTA (pH 8) added. RNA products were denatured at 92°C for 3 minutes and separated on 8M urea, 5% acrylamide gels. The dried gels were exposed to a phosphorimager screen and analyzed using QuantityOne (BioRad). To more precisely determine the size of the upstream cleavage products, aliquots from the T7 co-transcription/cleavage assays were denatured and separated on an 8 M urea, 7.5% acrylamide gel next to a DNA sequencing reaction primed from the -40 region of the m13 vector.

### Element sequences

Most R2/R4/*Dong* sequences can be found at GenBank and are under the following accession numbers: *Limulus* *polyphemus* , AF015814; *Nasonia* *vitripennis* R2B, AF090145; *Porcellio* *scaber* , AF015818; *Anurida* *maritima* , AF015815; *Bombyx mori*, M16558; *Forficula* *auricularia* , AF015819; *Triopscancriformis* , Eu854578; *Reticulitermes* *lucifugus* , GU949555; *Kalotermes* *flavicollis* , GU949558; *Ixodes* *scapularis* , ABJB010506112; *Ciona* *intestinalis* A, AB097121; *C* *. intestinalis* B, AB097122; *C* *. intestinalis* C (partial), AB097123; *C* *. intestinalis* D (partial), AB097124; *Rhynchosciaraamericana* , FJ461304; *Ascaris* *lumbricoides* R4, U29445; *Bombyx mori* Dong, L08889. R2 sequences from *Apis mellifera*, *Nematostella* *vectensis* , *Schmidtea* *mediterranea* , and *Taeniopygia* *guttata* as well as R8 sequences from *Hydra magnipapillata*, Baggins1 from *Drosophila melanogaster*, and L1Tc from *Trypanosoma cruzi* were obtained at Repbase (http://www.girinst.org). Composite R2 element sequences for *Tribolium* *castaneum* A, B, and C and *Tetranychusurticae* as well as the 5' end of the *C* *. intestinalis* C element (Stage DE, unpublished data) were assembled from the trace archive databases at GenBank (http://blast.ncbi.nlm.nih.gov/Blast.cgi) as described [24]. Briefly, trace reads with non-ribosomal sequence upstream of the R2 3' target site were used as a starting point. The consensus end of the newly elongated sequence was then used as the next blast query, and the procedure continued until 28S gene sequences upstream of the R2 5' target site were reached. These composite sequences as well as those for R2s from *Nasonia* *giraulti* and multiple Drosophila species are available at http://blogs.rochester.edu/EickbushLab/. Sequences for the three *T* *. castaneum* lineages were recently reported [25] and are similar except that the *T* *. castaneum* C element used here has an additional 500 nt at the 5' end.

### Phylogenetic relationships

The conceptual translations of the carboxyl-terminal half of the open reading frame starting at a conserved AF/YADD motif of the reverse transcription domain were used to create a sequence phylogeny. The R2 tree was rooted using the Baggins1 element from *D. melanogaster* and the L1Tc element from *T. cruzi* as outgroups. Sequences were aligned by ClustalW with default parameters, and the best tree generated using the Phylogenetic Reconstruction using the Neighbor Joining Method and uncorrected (“p”). The alignment can be found in Supporting Information, Figure S1.

## Results

### R2 5' junctions from seven species

Full-length R2 element sequences from *Nasonia* *vitripennis* (jewel wasp (Nv)), *Nasonia* *giraulti* (jewel wasp (Ng)), *Limulus* *polyphemus* (horseshoe crab), *Porcellio* *scaber* (sow bug), *Forficula* *auricularia* (earwig), *Anurida* *maritima* (springtail) and *Bombyx mori* (silkmoth) have been previously cloned and sequenced [22]. While these are all arthropod species, their R2s are from most of the known major lineages of R2 elements [16]. A single lineage of R2 element is found in five of these species, however, the two jewel wasp species have multiple divergent R2 families present in their genomes [26]. To ensure a full-length 5' junction had been recovered in each species as well as score the variation in these junctions, the 28S/R2 5' element junctions from these seven species were PCR amplified and an additional 3 to 7 clones sequenced. These sequences along with junctions published previously as well as junctions obtained from available trace archives indicated that unlike the extensive variation found with the R2 elements of Drosophila, the 5' junctions derived from jewel wasp (Nv), springtail, sow bug, and earwig were uniform (Figure 1B). Interestingly, all springtail 5' junctions had a 7 bp deletion of 28S rRNA sequences. All sow bug 5' junctions had a 4 bp deletion of the 28S gene and a 24 bp duplication of 28S gene sequences from upstream of the R2 target site. All earwig 5' junctions had a 22 bp duplication of 28S gene sequences from downstream of the R2 target site. The duplications in sow bug and earwig contained one and three nucleotide substitutions relative to the 28S sequence, respectively.

Multiple 5' junctions were observed in the remaining three species (Figure 1B). All 5' junctions in the silkmoth had a short (2 or 3 bp) deletion of the 28S gene, and approximately 10% of the junctions contained a direct duplication of upstream 28S sequences, 24-25 bp in length. Some of these junctions had additional non-templated nucleotides. Two junction types were observed in jewel wasp (Ng) with the most common junction (70%) having a 26 bp direct duplication of 28S gene sequences at the 5' end of the element. In horseshoe crab, over 90% of the junctions had a 21 bp duplication of upstream 28S sequences containing 3 nucleotide substitutions. These junctions varied upstream of this duplication in having either additional duplicated 28S sequences (7 or 113 nucleotides in length) or non-templated nucleotides. In summary, the R2 junctions in these additional arthropods clearly differed from those in Drosophila species where most junctions contain variable deletions of the 28S gene and/or non-templated additions but rarely duplications of 28S sequences [11,21].

### RNA self-cleavage occurs at different locations

**Figure 2:**
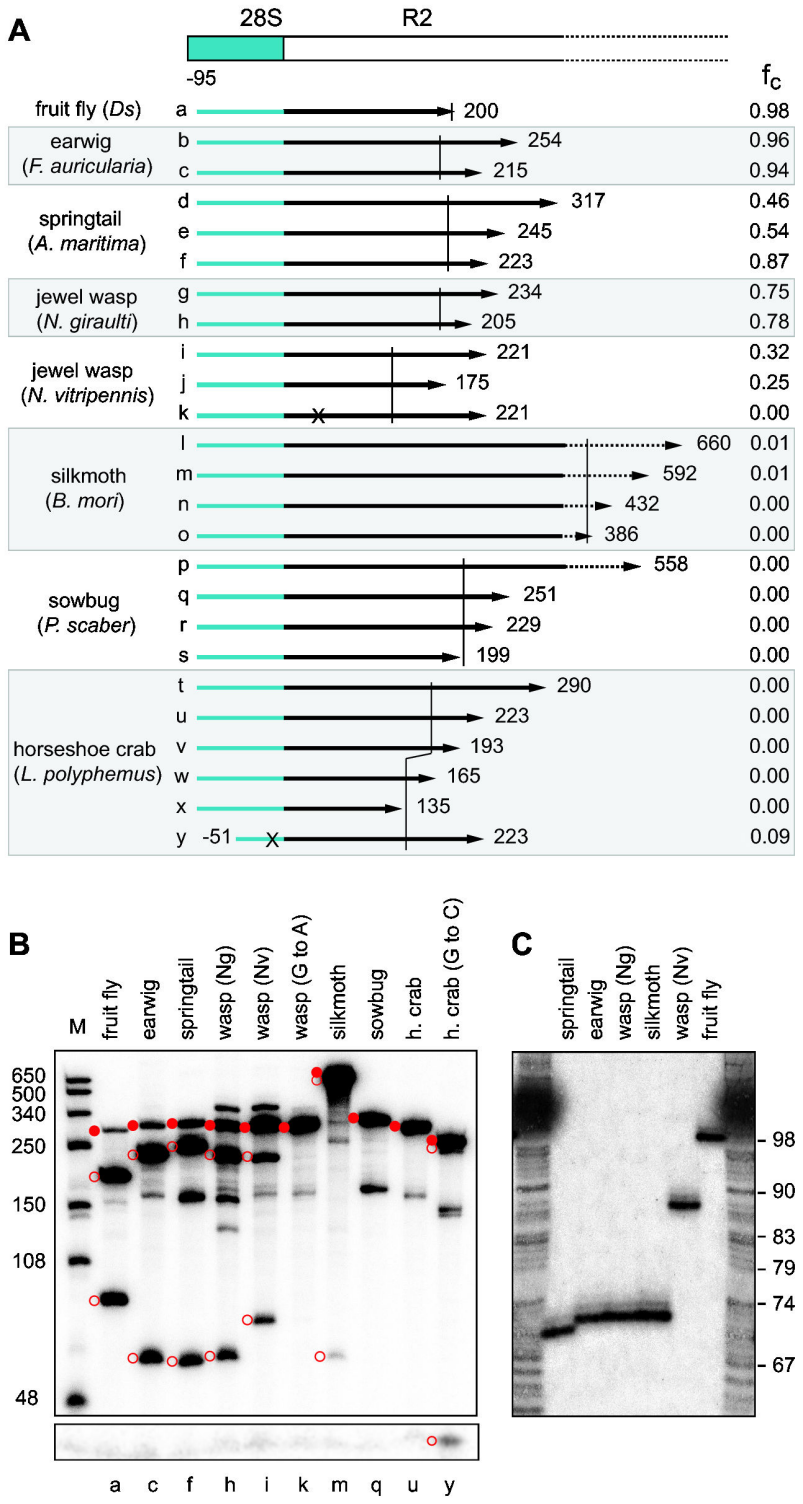
RNA comprising the 28S/R2 5' junction from many arthropods can function as a self-cleaving ribozyme. (A) Diagram of a generic 28S gene (blue box)/ R2 5' end (white box) junction. Arrows labeled *a* through *y* represent the *in* *vitro* generated RNAs tested for self-cleavage from the eight species listed on the left. *Ds*, *D* *. simulans* . RNAs (except *y*) begin at position -95 relative to the R2 insertion site and end at the noted nucleotide within the R2 element. RNA *k* has a 'G' to 'A' change in the active site of the ribozyme; RNA *y* has a generated 'G' to 'C' change to improve the P1 stem (see text). The short vertical lines demarcate the extent of the predicted ribozymes. To the far right of each arrow is the average fraction of RNA cleaved in at least two independent co-transcription/cleavage assays. (B) A 5% denaturing acrylamide gel showing the cleavage products for select RNAs in the co-transcription/self-cleavage assays. A longer exposure of the lower portion of the gel is shown to better visualize the short upstream cleavage product. The uncleaved RNA (solid red circles) and cleavage products (open red circles) are indicated. Unmarked bands are alternative, stable RNA structures. Lanes are labeled with the corresponding letter from panel (A). Lane M, RNA length markers with sizes indicated. (C) An 8% denaturing acrylamide gel showing the upstream cleavage products at single nucleotide resolution. RNAs are the same as in (B) except RNA from the silkmoth was from *S* *. cynthia* . DNA ladder corresponds to combined G, A, T sequencing reactions with nucleotide position from the -40 primer in the m13 vector shown to the right.

To test for RNA self-cleavage activity, DNA templates containing T7 promoter sequences were generated by PCR amplification of the cloned 28S/R2 5' junctions. Included in the analysis as a positive control was the ribozyme template previously characterized from the *D* *. simulans* R2 element [11]. The RNAs tested are diagrammed in Figure 2A and are labeled a through y. The transcription products were separated on 5% denaturing acrylamide gels, and the fraction undergoing self-cleavage (f_c_) quantified. The RNA products from select templates in these co-transcription/self-cleavage assays are shown in Figure 2B. High levels of RNA self-cleavage were detected for the 5' junction templates from four species: earwig (up to 96% self-cleavage), springtail (up to 87%), jewel wasp (Ng) (up to 78%), and jewel wasp (Nv) (up to 32%). Low levels of self-cleavage (~1%) were detected with the 5' junctions from silkmoth while self-cleavage was not detected for the sow bug or the wildtype horseshoe crab R2 5' junctions.

While the ability of R2s from other arthropods to self-cleave was expected, an unexpected finding was that the location of the cleavage differed from that in fruit fly. Earwig, springtail, jewel wasp (Ng), and silkmoth each had an upstream cleavage product that was ~25 nt shorter than that observed for fruit fly while the upstream product from jewel wasp (Nv) was ~10 nt shorter (Figure 2B, lower open circles). These length differences are not due to indels within the nucleotide sequence of the 28S gene from these species. Because self-cleavage occurs at the precise 5' end of the R2 element sequences in fruit fly [11], the shorter upstream products for the other species indicated that self-cleavage occurred upstream of the R2 junction.

### Ribozyme secondary structures

**Figure 3:**
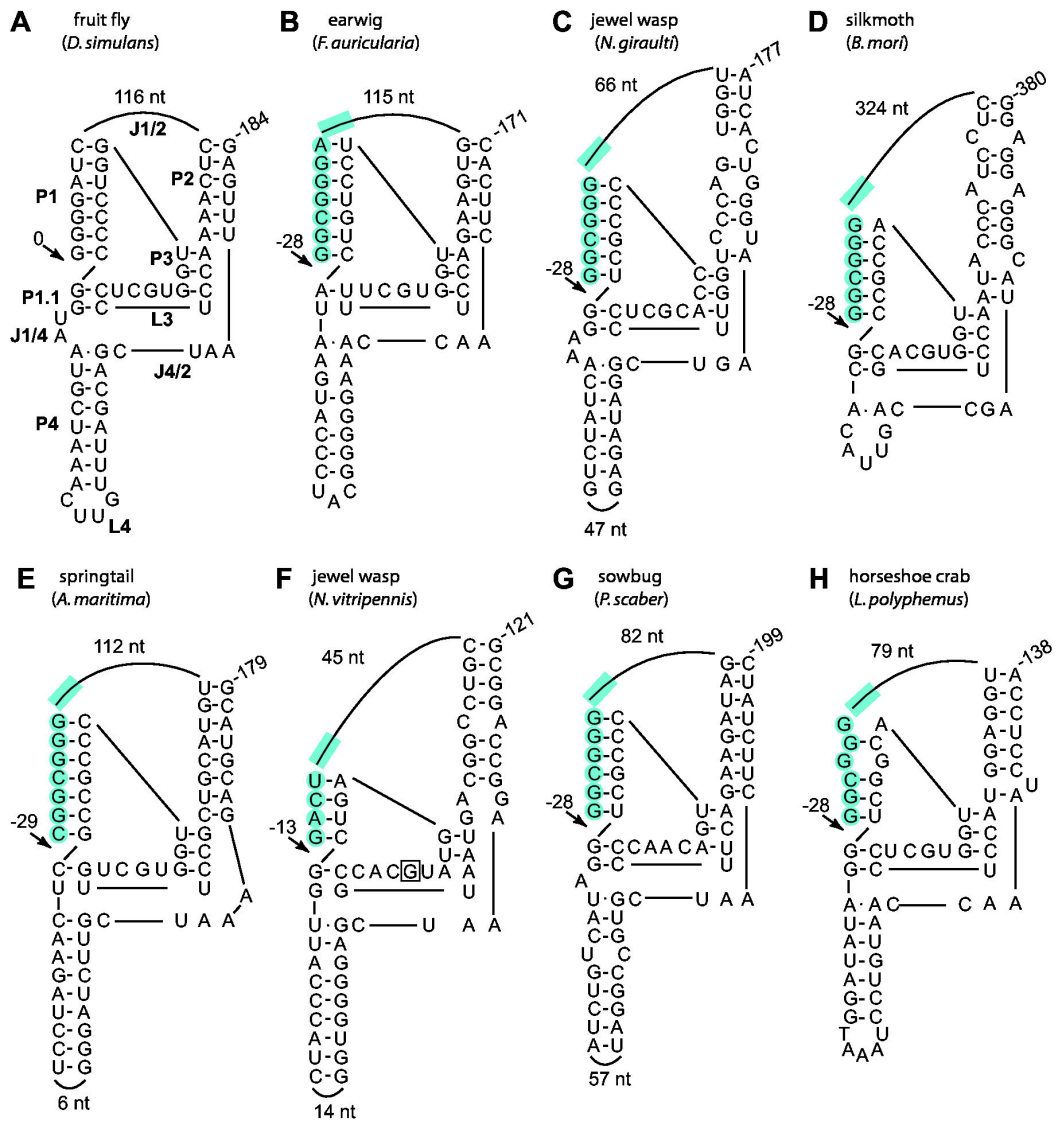
28S/R2 RNA sequences from divergent arthropods fold into structures similar to the ribozyme from Drosophila. (A) Diagram of the R2 ribozyme from *D* *. simulans* ; P, base-paired region, L, loop at end of a P region; J, nucleotides joining base-paired regions [11]. (B) through (H) Predicted secondary structures of the 28S/R2 junctions from seven arthropods. Only the number of R2 nucleotides in the J1/2 loop and the number of nucleotides in the L4 loop if it is large are shown. The presence of 28S gene sequences within the ribozyme is indicated with blue shading. Arrows indicate the observed or predicted R2 self-cleavage sites relative to the 3' R2 insertion site. A 'G' to 'A' nucleotide substitution at the boxed position in the *N* *. vitripennis* structure was observed to affect self-cleavage (Figure 2). Partial structures for earwig and horseshoe crab and an alternative P1 for silkmoth were previously predicted [12].

The different locations of R2 self-cleavage in the various arthropods were consistent with the secondary structures predicted for the RNA encompassing the 5' end of each element. In all seven species, structures similar to that determined for the R2 ribozyme of fruit fly could only be generated if 28S sequences were included in the 5' portion of the P1 stem (Figure 3A-F, shaded nucleotides). For earwig, jewel wasp (Ng), and silkmoth the structures predicted self-cleavage 28 bp upstream (-28) relative to the R2 3' insertion site in the 28S gene. The structure generated for springtail suggested self-cleavage 29 nt upstream while the structure generated for jewel wasp (Nv) suggested self-cleavage 13 nt upstream of the R2 3' insertion site. Electrophoresis of the RNA cleavage products described in the previous section at single nucleotide resolution confirmed these predicted self-cleavage sites (Figure 2C). The putative ribozyme structure presented for the R2 element from jewel wasp (Nv) could also explain why a single nucleotide substitution, a 'G' to 'A' in one tested 5' junction for this species (Figure 3F, boxed nucleotide), yielded no detectable self-cleavage (Figure 2B, lane k). This 'G' residue is highly conserved in the L3 region of the HDV-like ribozymes and forms a reverse wobble pair with a 'U' residue also in the L3 region [5]. This nucleotide substitution in HDV does not, however, completely eliminate cleavage [27] as it does in the *Nasonia* R2.

Sequences flanking the HDV ribozyme have been shown to effect cleavage activity by positively or negatively impacting the ability of the ribozyme to fold correctly [28,29]. The 3' end of the putative ribozyme sequence within the templates for each species is indicated with a vertical line in Figure 2A. The influence of downstream sequences on R2 cleavage was observed in only two species. First, the shortest template tested from springtail had a higher level of RNA self-cleavage than a template that was just 20 nucleotides longer (Figure 2A, e versus f). Second, in silkmoth only the longer templates resulted in observable self-cleavage (Figure 2A, l and m versus n and o) suggesting the downstream sequences may promote ribozyme folding in *B. mori*. Varying the amount of downstream R2 sequences did not promote cleavage in either the putative sow bug or horseshoe crab ribozymes (Figure 2A, constructs p and t).

While the sow bug generated RNAs had no detectable self-cleavage, the 28S/5' R2 junction sequences can be folded into the conserved ribozyme secondary structure with cleavage occurring at position -28 (Figure 3G). This putative sow bug ribozyme lacks the 'G' residue in the L3 catalytic region that was shown to be important in the jewel wasp (Nv) R2 ribozyme. The absence of an active ribozyme may indicate that this species (or at least the animal tested) does not have an active R2 element. Indeed, only a few copies of R2 were detected in this animal and sequencing revealed a disrupted ORF [22]. It is likely, therefore, that mutation(s) have also eliminated ribozyme activity in these dead elements.

In the case of the horseshoe crab tested, the R2 element sequenced did have an intact ORF and multiple different 28S/R2 5' junctions were recovered suggesting R2 activity. The ribozyme structure for the horseshoe crab R2 predicts cleavage 28 nt upstream of the R2 site (Figure 3H). All sequenced horseshoe crab R2 5' junctions have a 1 bp mismatch in the putative P1 stem. Such mismatches are uncommon in the P1 stem of HDV-like ribozymes and may explain the absence of *in vitro* self-cleavage activity in horseshoe crab. Indeed, a U-U mismatch in the P1 stem of an HDV-like ribozyme from the sunflower, *Helianthus* *annus* , as well as a C-C mismatch in the P1 stem from the mosquito, *Anopheles* *funestus* , have also been postulated to explain the absence of *in vitro* ribozyme cleavage activity except at elevated Mg^2+^ concentrations [30]. Therefore, a 'G' to 'C' substitution was introduced into the horseshoe crab RNA template at position -25 (Figure 2A, construct y), and a low level of cleavage (9%) was detected (Figure 2B, lane y). It is unclear why an R2 element with an apparently less active ribozyme dominates the R2 population in this species.

### R2 ribozyme in silkmoths

The very low activity of the ribozyme from the silkmoth R2 was of special interest because our biochemical studies of the R2 integration mechanism have been conducted with this element [17]. In addition, high levels of R2 are present in different geographic stocks of silkmoth suggesting the element is active [31]. The putative ribozyme structure associated with the *B. mori* R2 was unusual because it has an exceptionally long J1/2 loop and no P4 stem (Figure 3D). The P4 stem appears to stabilize HDV-like ribozymes, however, it is not required for HDV activity [32,33] and an HDV-like ribozyme with only 5 nucleotides instead of a P4 stem has been found in the abalone, *Haliotis* *discus* [30]. To provide phylogenetic support for the proposed RNA secondary structure, R2 elements in four additional species of moths from the same superfamily of moths as *B. mori* (Bombycoidea) [34] were analyzed: *Samia cynthia*, *Callosamiapromethea* , *Coscinocera* *hercules* , and *Saturnia* *pyri* . DNA templates corresponding to the 28S/R2 5' junction [23] were generated for each species, and the RNA products quantitated after co-transcription/self-cleavage reactions. The *in vitro* generated RNAs from all four additional species were found to self-cleave at a higher level than that observed for *B. mori*, approximately 10% for *S* *. pyri* and *C* *. hercules* and 25% for *C* *. promethea* and *S* *. cynthia* [Eickbush DG, unpublished data].

**Figure 4:**
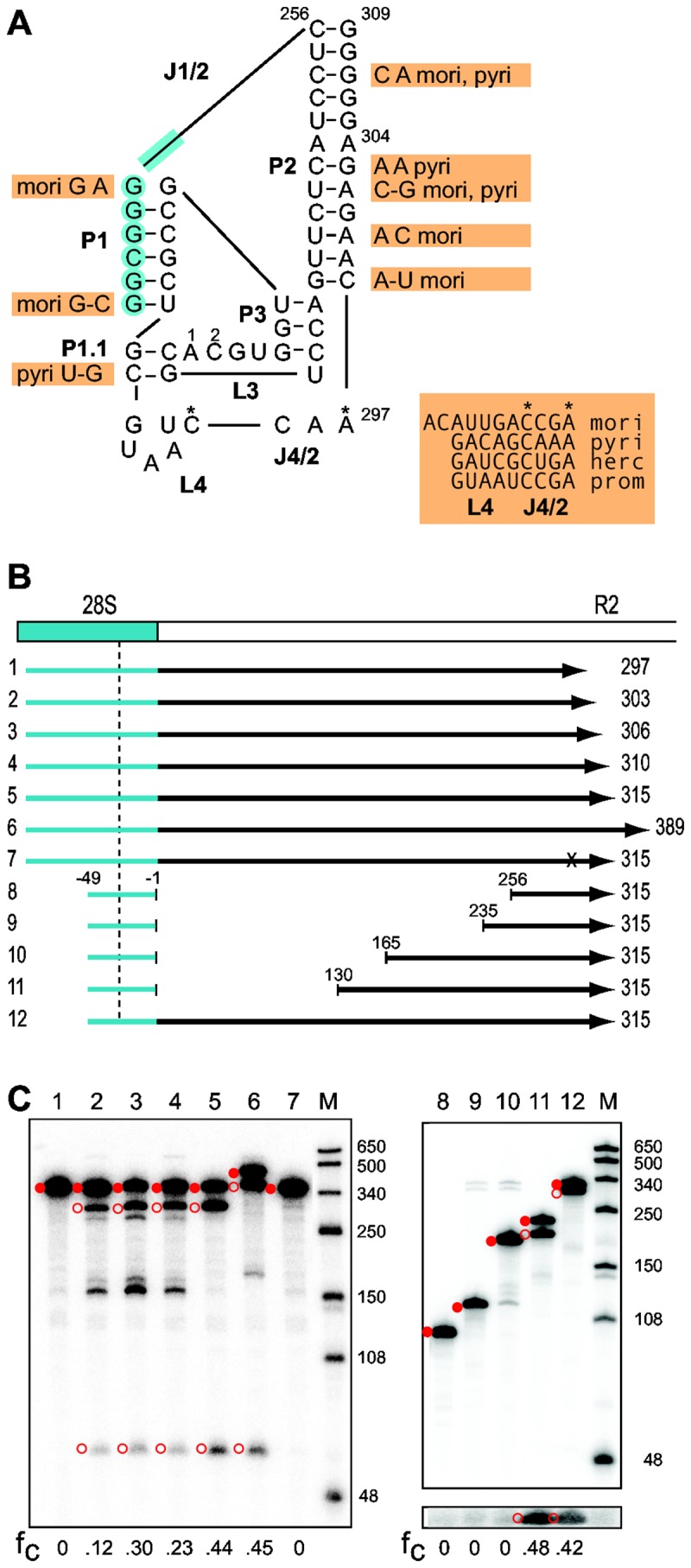
Comparison of the R2 ribozyme from 5 silkmoth species. (A) Secondary structure of the putative ribozyme encoded by the R2 element of *S* *. cynthia* . Blue shading indicates nucleotides from the 28S gene. Indicated next to the *S* *. cynthia* sequence are differences in the R2 sequences from 4 silkmoth species (nucleotides in orange boxes). The entire sequence of the largely unconserved L4- J4/2 region is presented for each species. Two species contained nucleotide differences in the otherwise conserved L3 (residue 1 is a 'U' in *S* *. pyri* , residue 2 is an 'A' in *C* *. hercules* ). The J1/2 loop length varied from 216 nt in *S* *. pyri* to 498 nt in *C* *. hercules* *mori*, *B. mori*; herc, *C* *. hercules* ; prom, *C* *. promethea* ; *pyri*, *S* *. pyri* . (B) Diagrams of the 28S/R2 RNAs from *S* *. cynthia* tested for self-cleavage as described in Figure 2. The vertical dashed line indicates the predicted upstream cleavage site at -28. Negative numbers indicate position in the 28S gene relative to the R2 insertion site; positive numbers indicate position in the *S* *. cynthia* R2. The *x* in RNA 7 represents a 'U' substitution for the putative catalytic 'C'. Templates 8-11 have varying amounts of the J1/2 loop removed. (C) 5% denaturing acrylamide gels showing the products from the co-transcription/self-cleavage assays. Lane numbers correspond to the RNAs in panel (B). The uncleaved RNA (solid red circle) and self-cleavage products (open red circles) are indicated. Lane M, RNA length markers with sizes indicated. For RNAs 8-12, a longer exposure of the lower portion of the gel is shown to better visualize the 21 nt upstream cleavage products. The fraction of the synthesized RNA undergoing self-cleavage (fc) is shown at the bottom of each gel.

The 28S/R2 5' junction sequence of all four species could be folded into a secondary structure similar to that proposed for the *B. mori* ribozyme (Figure 4A). As previously found for Drosophila species [11], the fifteen nucleotides corresponding to the P1.1, P3, and L3 regions show high sequence identity. However, unlike the extensive compensatory differences in the P1 stem of the fruit fly ribozymes, the silkmoth P1 stems were conserved due to constraints on the 28S gene sequence that makes up the 5' end of the ribozyme. The J1/2 loops ranged from 216 nt in *S* *. pyri* to 498 in *C* *. hercules* and contained only small regions of sequence identity between species. The P4 stem was absent in all five species and with the exceptions of the putative catalytic 'C' and a constrained 'A' residue little sequence similarity was found in the region encompassing L4 and J4/2 (Figure 4A).

To further define the silkmoth ribozyme, the self-cleavage activity of the *S* *. cynthia* ribozyme was tested in additional co-transcription/self-cleavage assays (Figure 4B). Self-cleavage was not detected when the RNA ended at position 297 (Figure 4C, lane 1), the junction of the J4/2 and P2 regions of the proposed ribozyme structure (Figure 4A). RNA that ended at position 303 in the middle of the long P2 stem had 12% self-cleavage (Figure 4C, lane 2). An additional three or seven nucleotides in the RNA doubled the level of self-cleavage (Figure 4C, lanes 3 and 4). Extending the RNA five or eighty nucleotides beyond the P2 stem further increased the level of self-cleavage to over 40% (Figure 4C, lanes 5 and 6). Substitution of a 'U' for the 'C' at position 294 (Figure 4A, C*) eliminated self-cleavage confirming it as the catalytic residue (Figure 4C, lane 7). Finally, to test whether sequences in the large J1/2 loop were unnecessary for ribozyme activity as had been found for the fruit fly enzyme [11], the ~ 250 nt R2 portion of the J1/2 loop was removed. Self-cleavage was not detected (Figure 4C, lane 8). To determine what region was required for cleavage, increasingly longer segments starting from the 3' end of the loop were added back to the ribozyme (Figure 4B). RNAs with 20 or 90 nucleotides of the loop showed no detectable self-cleavage (Figure 4C, lanes 9 and 10); however, RNA with 126 nucleotides had a level of self-cleavage comparable to the control template (Figure 4C, lanes 11 and 12). It will be interesting to determine how this 35 nt region within the J1/2 loop contributes to the structure of the ribozyme.

### R2 ribozyme cleavage sites in additional species

**Figure 5:**
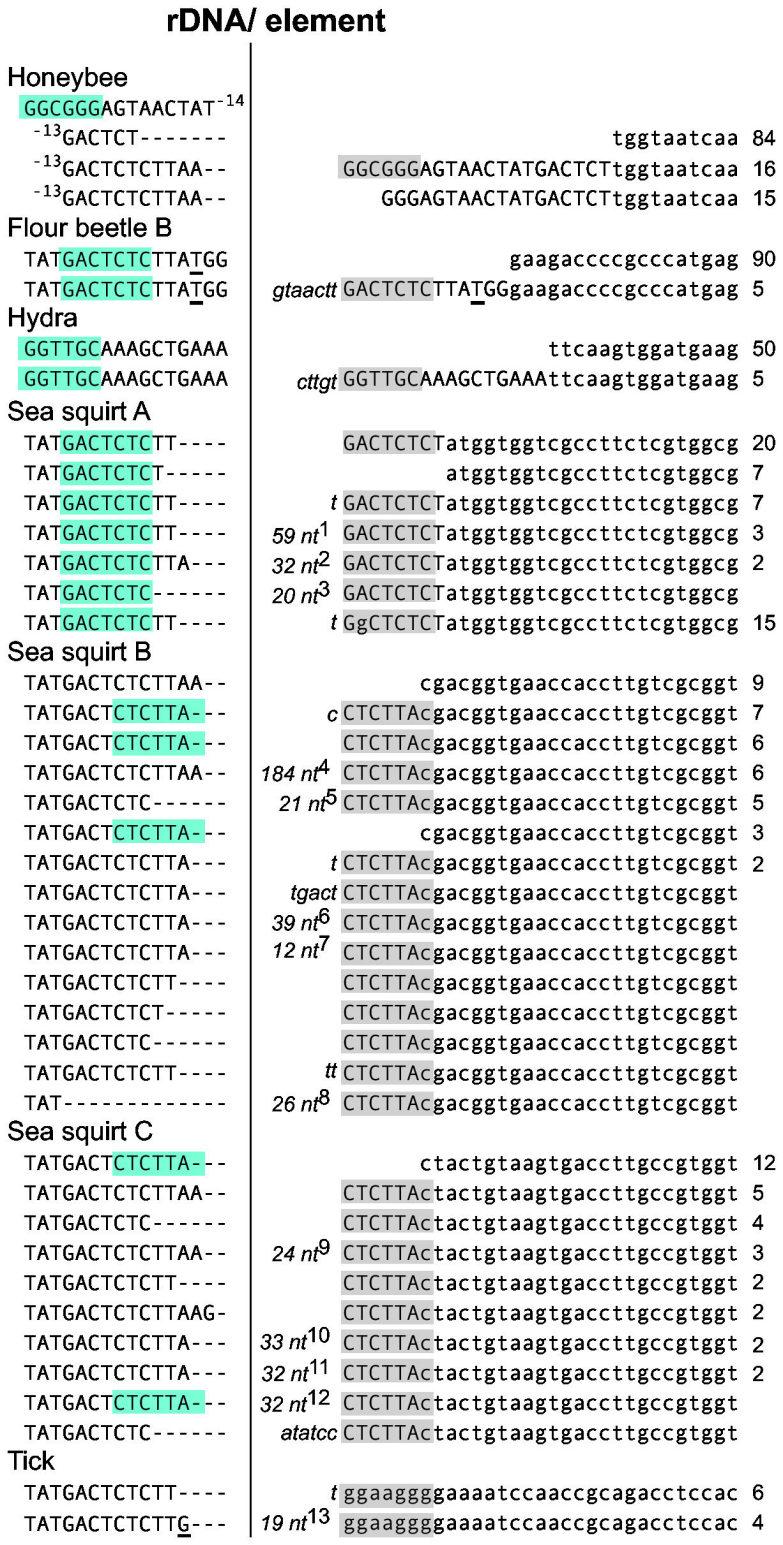
Variation in the 28S/R2 5' junction reflects cleavage site position. R2 sequences, 28S sequences, and non-templated sequences are as described in Figure 1. Single nucleotides in the flour beetle and tick 28S genes that differ from the consensus arthropod 28S gene sequence are underlined. All sequences are derived from the trace reads of genomic sequencing projects. The number of trace reads containing the same junction sequence is indicated to the far right. Nucleotides that could form the 5' end of the predicted P1 stem of the ribozyme (Supporting Information, Figure S2) are boxed in blue if from the 28S gene or in gray if from R2. Two regions can potentially anneal because of 28S duplications associated with some junctions. Sequences not presented in the figure: ^1^ AGTAACTATGACTCTCTTTGAGTAACTATGACTCTCTTTGAGTAACTATGACTCTCTTT; ^2^ TGAACTCTCTATGGTGGTCGCCTTCTCGTATG; ^3^ GTAACTATGACTCTCTCTTT; ^4^cyclin A-like, putative template jump [18]; ^5^ GGGAGTAACTATGACTCTCTT; ^6^ TGACTCTCTTATTTATGACTCTCCTATGACTCTCTTATT; ^7^ GAGACCAACTTA; ^8^ GGCGGGAGTAACTCTGACTCTCTTTT; ^9^ CTATGACTAACTATGACTCTCTTT; ^10^ GCTAACTCTGACTCTCTTAGCTGACTCTCTTTT; ^11^ TGACTCTCGGCGGGAGTAACTATGACTCTCTT; ^12^ CTACTGTATGACTCTCTTACTACTGTATGACT; ^13^ TCTTGTCTCTTGTCTCTTG.

To further sample the range of structures and cleavage sites associated with the R2 ribozyme, HDV-like ribozyme structures were generated for 18 R2 elements found in other arthropod as well as non-arthropod species (Supporting Information, Figure S2A). The R2 ribozyme structures from these 13 species predict additional examples of self-cleavage at the 5' end of the element or in the 28S sequences 9 to 36 nucleotides upstream of the insertion site. For the nine species in which trace reads are available from genomic sequencing projects, we could again correlate sequence variation at the 5' end of the R2 elements with the different R2 self-cleavage sites. For all R2 elements whose ribozymes were predicted to cleave within the 28S sequences, the majority of the R2 5' junctions were uniform. In the case of honeybee, flour beetle B, and hydra, 5' junctions were also found which contained tandem duplications of upstream 28S sequences of a length consistent with the location of the predicted cleavage site (Figure 5). For example in flour beetle B, cleavage was predicted at -13 and about 5% of the R2 5' junctions contain a tandem duplication of these 13 nucleotides.

On the other hand R2 lineages in sea squirt and tick, whose ribozymes were predicted to cleave at the R2 junction, had many 5' junctions with small deletions of upstream 28S gene sequences and/or nucleotide additions (Figure 5). The sea squirt junctions differ from those found in fruit fly in that most have duplicated 28S gene sequences at the 5' end of the element and long stretches of added nucleotides, frequently with sequence identity to upstream 28S sequences. The tadpole shrimp ( *T* *. cancriformis* ) has been reported to have junctions with variable deletions of upstream 28S gene sequences [35] consistent with the prediction of self-cleavage at the R2 junction.

### Cleavage site distribution in the R2 element phylogeny

**Figure 6:**
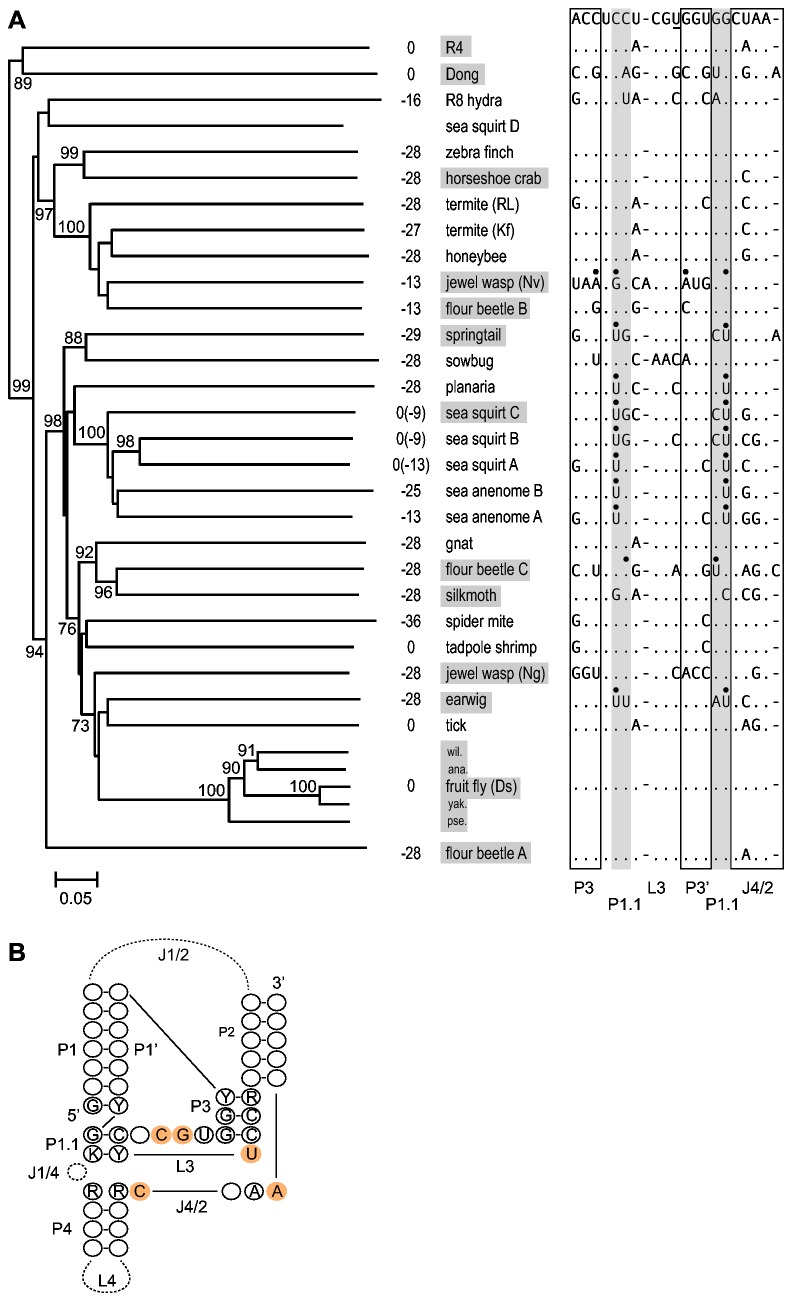
R2 element phylogeny and its correlation with cleavage location and ribozyme sequence. (A) Best tree based on the neighbor-joining method and rooted using the retrotransposons Baggins1 and L1Tc. Most elements are listed in Figure 2 and Supporting Information, Figure S2. Additional fruit fly elements include: *D* *. willistoni* (wil.), *D* *. ananassae* (ana.), *D* *. yakuba* (yak.), and *D* *. pseudoobscura* (pse.). Bootstrap values above 70% are shown. Numbers left of the elements are predicted or demonstrated (boxed elements) self-cleavage locations relative to the R2 insertion site. The 5' junction cleavage site agrees with previous predictions for sea squirt A, tick, and R4; however, the tadpole 5' end is shifted 1 nucleotide and 28S cleavage sites are suggested for zebra finch, gnat, and both termites [12]. The 5' end of the sea squirt D element could not be folded and may be incomplete. To the right of each element are sequence differences in the ribozyme active site relative to the R2 consensus sequence (top line). The underlined 'U' in this consensus is a 'C' in the HDV antigenomic ribozyme. Black dots indicate nucleotides that disrupt P3 or P1.1 stem pairing. (B) Consensus structure of the R2 ribozymes based on 26 elements (additional fruit fly elements were not included) in panel (A). Length differences and mismatches in the P1, P2, and P3 stems are described in the text. Nucleotides present in at least 75% of the R2 ribozymes are indicated. Invariant nucleotides are shaded orange. The sow bug ribozyme, which does not show activity *in* *vitro*, has 'C' to 'A' and 'G' to 'A' differences at the conserved sites in L3. Y= U or C; R= A or G; K= G or U.

Multiple lineages of R2 have been differentially maintained in animal lineages [15,16,36]. As a result, the phylogeny of R2 elements and the phylogeny of the host organisms are frequently not congruent [14,37]. Figure 6A shows the phylogeny of 31 R2 elements as well as 2 elements from the related R4 clade. The phylogenetic tree was constructed using the ~480 amino acid residues located carboxy terminal to the conserved AF/YADD motif found in the reverse transcriptase of all non-LTR retrotransposable elements. The R2 phylogeny is largely congruent with previously reported R2 phylogenies that are composed of different subsets of R2 and related non-LTR retrotransposons [15,25,38].

The proposed location of ribozyme self-cleavage for each R2 element is shown to the right of the branch in the phylogeny in Figure 6A. Because flour beetle A represented a divergent R2 lineage [22] not previously tested and *Dong* represented a second example in the R4 lineage [39,40], their self-cleavage activities along with those of two other flour beetle elements were confirmed in co-transcription assays (Supporting Information, Figure S2B). The most frequent location of self-cleavage is in the 28S gene at position -28 (Figure 6A). This cleavage site occurs in many divergent R2 lineages, including the zebra finch, planaria, silkmoth, and flour beetle A elements. This suggests a model in which cleavage shifted from the 5' end of the element, as seen in other non-LTR retrotransposons (e.g. R4, *Dong*, baggins, L1Tc), to position -28 early in R2 evolution. We propose nucleotide substitutions in the element portion of the P1 stem in different R2 lineages likely resulted in short shifts in this upstream cleavage site in the sea anemone B (cleavage at -25), termite Kf (cleavage at -27), and springtail (cleavage at -29) elements. A model which further elaborates possible paths which shift the cleavage site, including back to the 5' junction, is presented later.

### The R2 ribozyme

Because R2 elements have been vertically inherited with the genomes of their animal hosts for 100’s of millions of years, R2 offers a unique opportunity to follow the long-term evolution of and flexibility afforded to its ribozyme. Figure 6B shows the consensus secondary structure for the R2 ribozymes with all nucleotides present in at least 75% of the R2 elements indicated. The P1 stem is typically 6 or 7 bp in length with about one fourth of the elements containing 1-2 mismatches at the top of this stem. The P2 stem is at least 5 bp in length, but about one third of the elements contain one or more mismatches in the first 5 pairs of this stem. The P4 region is highly variable in R2 with some ribozymes having too few nucleotides to form a structure, some having little stem structure, and others having stems with extensive base pairing. The J1/4 region is also variable and can contain 0-2 nucleotides. There is little conservation of the primary sequence of the R2 ribozyme active site except for the catalytic 'C' and 4 other rigidly conserved nucleotide positions. Similar sequence flexibility has been found in studies of the HDV ribozyme [3,7,8] as well as other HDV-like ribozymes [30].

These comparisons suggest that although all R2 ribozymes have adapted to the same function (cleavage from the 28S gene) and, thus, most nucleotide differences probably reflect near neutral fluctuations, the R2 ribozymes contain much of the flexibility observed in other HDV-like ribozymes that have been incorporated into different genetic elements [10,12]. Indeed, the 4 nucleotide P1, mismatch in P3, and the spacing of the conserved nucleotides in L3 of the jewel wasp (Nv) (Figures 2B, 3F) deviate substantially from the structure of most HDV-like ribozymes [30].

## Discussion

Our previous studies have shown that self-cleavage of the R2 sequences from a 28S/R2 co-transcript in multiple species of Drosophila was at the precise 5' end of the R2 element [11,20]. Cleavage at the R2 5' junction coincides with the cleavage site associated with the ribozymes from other non-LTR retrotransposon clades- R4 (R4 [12]; *Dong*, Figure 6A), L1 [13], RTE [12]. In this report, analyses of the self-cleavage activity of diverse R2 elements indicated that in most species cleavage is within the 28S rRNA gene from 9 to 36 nt upstream of the R2 5' junction. This is the first report indicating that non-element sequences can become incorporated into the HDV-like ribozyme structure.

The variation in location of the R2 ribozyme cleavage helps to explain the extensive differences found at the 5' junctions of R2 elements from different species [22]. In Drosophila species, most junctions contained variable deletions of the 28S target site and variable additions of non-templated nucleotides [21,24]. However in most animals, R2 5' junctions within a species showed little variation except for direct duplications of the 28S gene of a characteristic length for each species (Figures 1, 5). Whether the R2 elements generate uniform or variable 5' junctions correlated well with the location of the RNA cleavage site.

**Figure 7:**
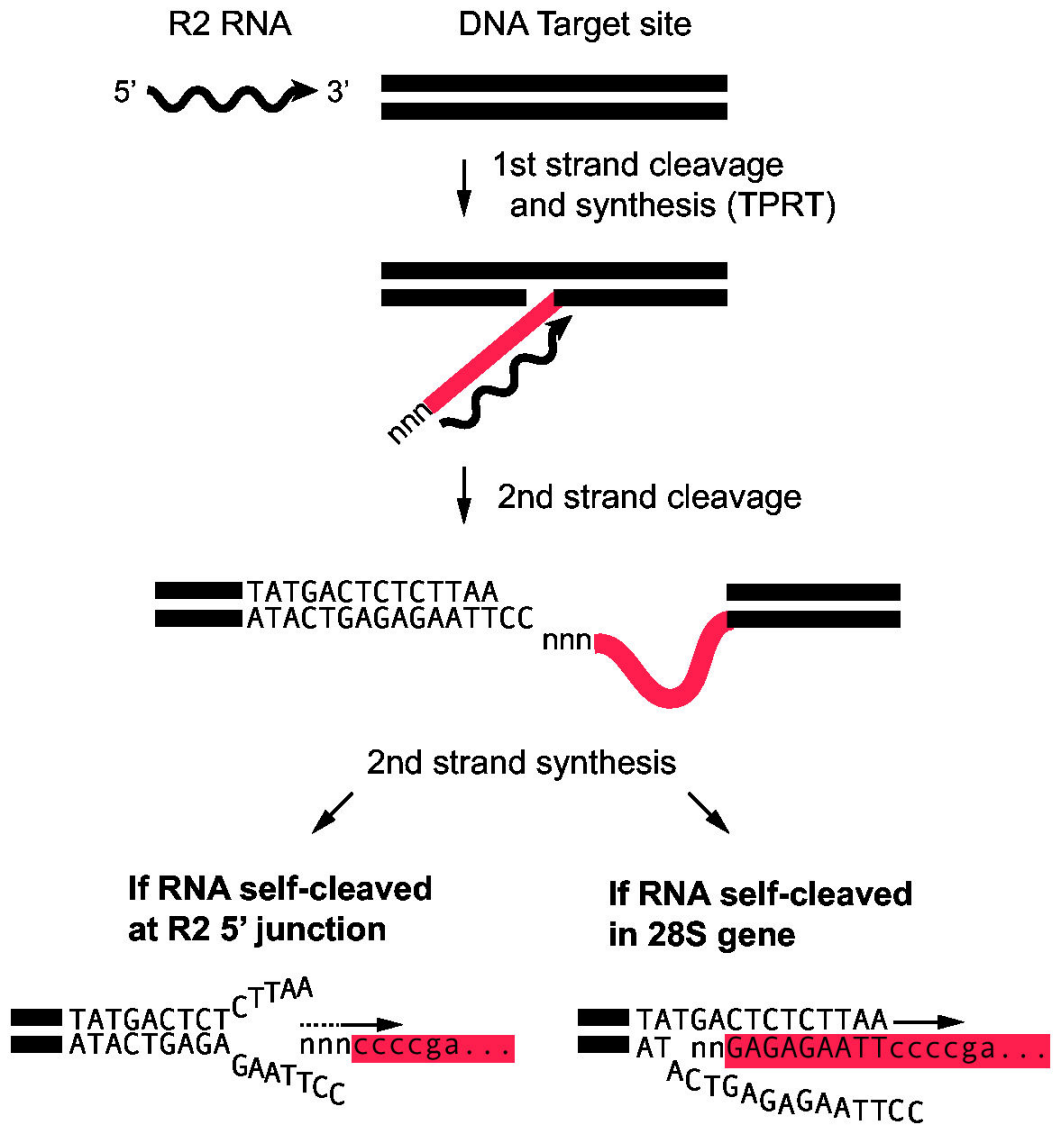
Models for the priming of second strand DNA synthesis in an R2 retrotransposition reaction. The initial steps of R2 integration are well characterized [41] and believed to be the same for all species. The R2 protein (not shown) recognizes the 3' UTR of the R2 RNA, binds to the 28S DNA target site, and cleaves the bottom strand. This cleavage site is used to prime first stand DNA synthesis which is referred to as target primed DNA synthesis, TPRT (cDNA, red line). Additional/non-templated nucleotides were shown to be added to the cDNA as the polymerase runs off an RNA template [42]. Priming of second strand DNA synthesis is proposed to differ between species based on whether the R2 RNA underwent self-cleavage at the R2 5' junction or upstream in the 28S gene. In the former, the R2 reverse transcriptase is able to use regions of microhomology of the cDNA with the DNA target upstream of the insertion site to initiate second strand DNA synthesis. This priming can involve the extra nucleotides added to the cDNA strand and can give rise to different length deletions of 28S sequences (lower left). In those animals where RNA self-cleavage is in the upstream 28S sequences, a heteroduplex between the cDNA and the target DNA is predicted to stabilize the integration intermediate resulting in a higher frequency of precise 5' junctions (lower right).

Figure 7 illustrates how the differences in the location of ribozyme cleavage, and thus the sequences at the 5' end of the RNA transcript used for retrotransposition, can be integrated into our model of R2 insertion [41]. The greatest uncertainty in the R2 integration model is the mechanism by which second strand DNA synthesis is initiated. In those cases where ribozyme cleavage is at the junction of the R2 element and the rRNA gene, there is no sequence identity between the first strand of DNA generated by the reverse transcriptase (i.e. the cDNA strand) and the upstream 28S gene sequence of the target site (Figure 7, bottom left). Second strand DNA synthesis is postulated to initiate by the R2 reverse transcriptase using regions of microhomology with the DNA strand upstream of the insertion site. This priming by microhomology usually involves extra nucleotides added to the cDNA strand as the reverse transcriptase runs off an RNA template [42]. Because these extra nucleotides are random, the microhomologies possible in each integration event will vary which gives rise to different length deletions of 28S sequences and/or non-templated nucleotide additions (Figure 7, bottom left).

On the other hand, extensive annealing between the cDNA strand and the upstream 28S target sequences is possible when ribozyme self-cleavage is within the upstream 28S sequences (Figure 7, bottom right). The formation of a stable heteroduplex gives rise to the precise start of second strand synthesis and in turn uniform 5' junctions within a species. In this scenario, any 28S/R2 junction variation generated during an aberrant integration event or by mutation can subsequently be included in the RNA template and propagated. This could explain why many R2 elements in *B. mori* contain an unusual 3 bp deletion of the 28S gene, why springtail elements contain a uniform 7 bp deletion, and why earwig elements contain 28S sequences from downstream of the insertion site at the 5' end of all R2 elements (Figure 1B).

Direct support for the model in Figure 7 can be found in the analysis of 5' junctions generated in an *in vivo* R2 integration system in Drosophila [43]. In these experiments RNA containing upstream 28S sequences attached to an R2 mini element (5' and 3' UTRs only) were injected into *D. melanogaster* embryos along with the R2 protein from *B. mori*. Analysis of the R2Bm integrants revealed that over 80% of these junctions were precise (no deletions of 28S sequences or non-templated nucleotides at the 5' end). In contrast, when R2 mini RNA without the upstream 28S sequences was injected, none of the 5' junctions were precise. Similar models for the formation of a heteroduplex between the cDNA strand and the DNA template have also been hypothesized during initiation of second strand synthesis for the non-LTR retrotransposons L1 [44] and R1 [24].

The presence of upstream 28S sequences in the processed co-transcript does not, however, guarantee uniform junctions. Direct (tandemly) duplicated rRNA gene sequences, varying from 13 to 28 nucleotides in length, are found at the 5' end of some copies in silkmoth, jewel wasp, honeybee, flour beetle, and hydra (Figures 1B, 5) and suggest that integration can also occur at the 5' end of the cDNA without the formation of a cDNA/upstream DNA heteroduplex. Priming of second strand DNA synthesis in these cases would be by way of microhomologies, thus, such integrations would be predicted to frequently contain upstream rDNA deletions or non-templated nucleotides between the two repeats as were observed in silkmoth, flour beetle B, and hydra (Figures 1B, 5).

While RNA cleavage within the 28S gene gives rise to more uniform junctions than cleavage at the 5' end of the element, whether upstream cleavage also gives rise to more efficient retrotransposition cycles can only be speculated. Critical factors are likely to be the manner in which the R2 protein is bound to the target sequence upstream of the insertion site and the level of sequence homology necessary to initiate second strand synthesis. At one extreme, in some species the bound R2 protein could enhance DNA unwinding and annealing of 28S gene sequences in the cDNA to the target site to initiate second strand synthesis. At the other extreme, in some species the bound R2 protein may prevent target site unwinding and, thus, the polymerase is adept at using microhomologies to initiate second strand synthesis. Unfortunately, direct studies of the R2 protein bound to the target site have only been conducted with one R2 element, the silkmoth *B. mori* [19,45].

### Evolution of the ribozyme catalytic site

The location of cleavage in HDV-like ribozymes is determined by the ability of an ~7 nt sequence, P1, to anneal to the ~7 nts, P1', immediately downstream of the critical L3/P3 sequences of the active site (Figure 6B). In the R2 ribozymes, these two segments are separated by as little as 23 nucleotides (sea anemone A) to over 700 nucleotides (honeybee). P1 stem formation in R2 most frequently involves sequences starting 28 bp upstream of the R2 target site (Figure 6A). Cleavage at this site may have occurred early in R2 evolution and is now used in many R2 lineages either because the region of the 28S gene from -28 to -22 is GC rich and can form a highly stable P1 stem or because a 28 nt extension is an ideal length to generate a heteroduplex between the cDNA and the target site (Figure 7). However, RNA cleavage in other lineages of R2 was observed at alternative positions upstream of the insertion site. Because the sequence of the 28S gene upstream of the R2 insertion site is nearly identical in all animals, these shifts in cleavage location are not driven by differences in the sequence of the 28S gene. We proposed above that random changes in the P1' sequence will on occasion give rise to pairing at other 28S gene locations and, thus, shift the cleavage site. Furthermore, the nucleotide sequence at a 28S/R2 5' junction resulting from deletions and/or additions will on occasion approximate the cleavage site, therefore, changes in the P1' can potentially give rise to pairing at this *de novo* P1.

**Figure 8:**
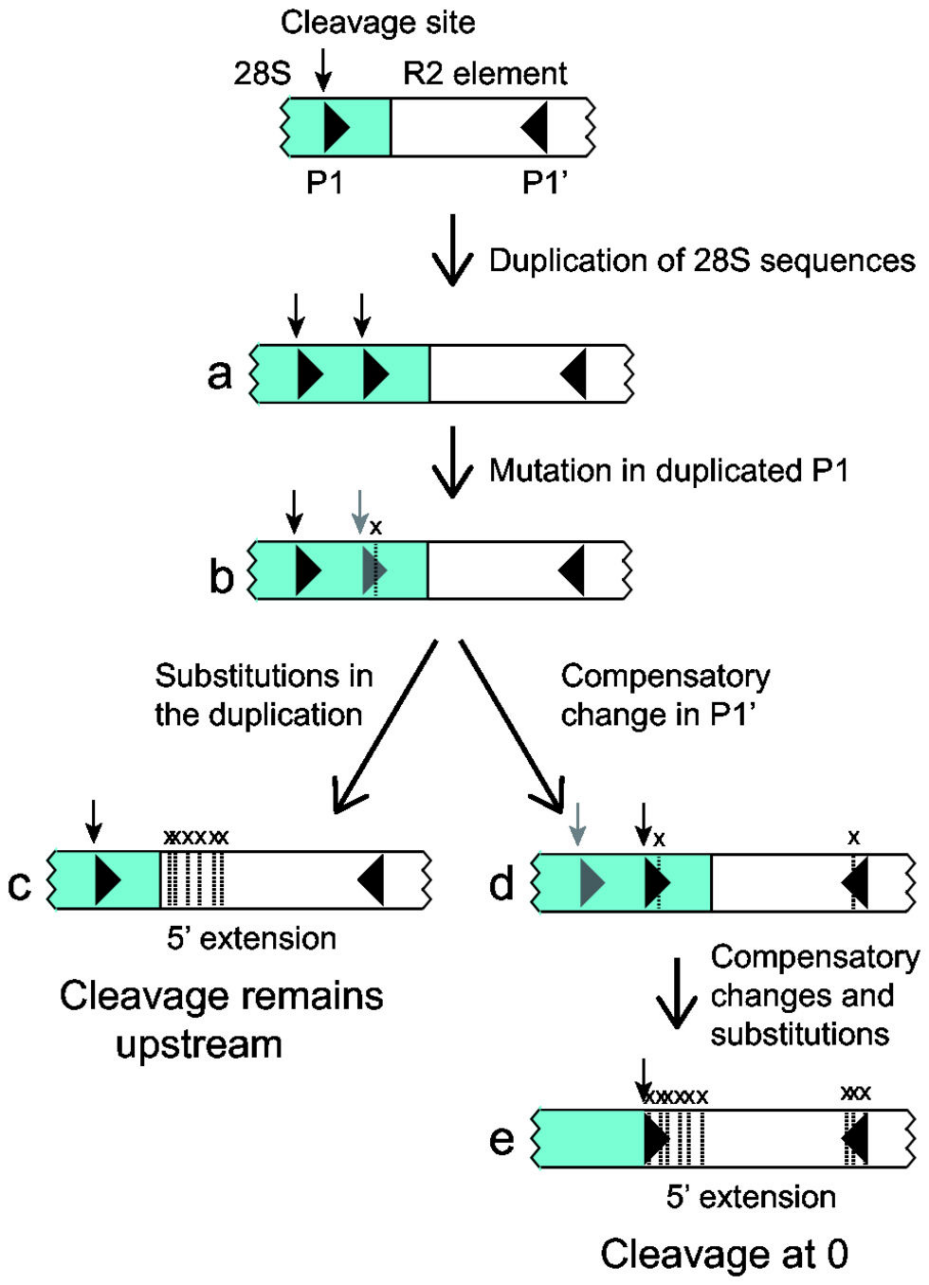
Model for how target site duplications can shift the R2 cleavage site. Diagramed is a typical 28S/R2 5' junction with 28S sequences in blue. The 28S upstream ribozyme self-cleavage site is indicated with a vertical arrow. Indicated with triangles are the upstream (P1) and downstream (P1') segments of the P1 stem of the ribozyme. Occasionally, the product of an R2 integration event is the duplication of 28S sequences at the new junction (a). A mutation in the internal P1 sequences in this junction will result in a higher likelihood of self-cleavage at the upstream site than at the downstream site, black and gray symbols respectively (b). Additional mutations in the duplication will eventually result in loss of sequence identity to the 28S gene (c). The nucleotide substitution in (b) can on occasion be compensated by a nucleotide substitution in P1', and self-cleavage will now more likely occur at the internal P1 (d). As further compensatory mutations accumulate, the duplication will lose identity to the 28S gene, and cleavage has shifted to the 5' end of the R2 element (e). Both scenarios give rise to a 5' extension of the R2 element.

The tendency for the R2 machinery to generate direct duplications of 28S sequences at the 5' end of the insertion also provides the opportunity to alter both the location of the RNA cleavage site as well as modify the 5' end of the R2 element (Figure 8). When an R2 insertion with a direct duplication of the target site is transcribed, self-cleavage can presumably occur at either of the two P1 sequences (diagram a). Cleavage at the internal P1 will eliminate the 28S duplication in subsequent retrotransposition cycles. However, if a substitution occurs in the duplicated P1 sequence (diagram b), self-cleavage will predominantly occur within the original 28S sequences, and the duplication will be propagated in subsequent cycles of retrotranspositions. Because nucleotides within the extension are not under constraint, they can rapidly accumulate substitutions that eventually eliminate any evidence that they were originally derived from the 28S gene. In this scenario, the 5' end of the R2 element has been extended, however, cleavage has remained in the upstream 28S sequences (diagram c). The large sizes of the J1/2 loop in some R2 elements may be the result of such repeated extensions of the 5' end.

Alternatively, the R2 element shown in Figure 8, diagram b can undergo a compensatory substitution in P1' which establishes more efficient RNA cleavage within the duplicated 28S sequences (diagram d). The sea squirt R2 B and C elements appear to be examples of such an event. Most copies of these elements contain a short duplication of the 28S gene, and the P1 stem that forms with the duplicated sequence is 1 nt longer and, thus, more stable than the stem that forms with the original 28S sequence (Figure 5). Further compensatory changes in the P1 and P1' motifs will eventually eliminate evidence that the 5' extension of the R2 element once corresponded to 28S sequences. Again the 5' end of the R2 element has been extended, but in this scenario cleavage has shifted to position 0 (Figure 8, diagram e).

### Evolution of HDV-like ribozymes

One of the most surprising findings from the original discovery of the ribozyme in *D* *. simulans* R2 elements was that, in addition to the same secondary structure and in spite of the exceptional flexibility in sequence observed in *in vitro* studies of the HDV ribozyme activity, the active site nucleotides of the *D* *. simulans* ribozyme showed remarkable sequence similarity to that of HDV [11]. The complex structure of the HDV ribozyme, which unlike simpler self-cleaving ribozymes has not been regenerated by *in vitro* selection experiments [46,47], has led to the suggestion that this ribozyme was unlikely to have evolved multiple, independent times [30]. Thus the ribozymes associated with HDV and non-LTR retrotransposons were suggested to have a common ancestor [12]. While the question of their ancestry will be difficult to resolve, the survey of R2 ribozyme variation reported here provides additional insights into the previously observed similarity with HDV. Most R2 ribozymes have multiple nucleotide differences in their active site relative to the HDV sequence (Figure 6A). There are, however, examples in addition to fruit fly in which the active site of the R2 ribozyme (zebra finch, gnat, flour beetle A) is more similar to that of HDV than to related R2 lineages. The discord between the R2 ribozyme sequences and the R2 phylogeny based on the sequence of the ORF is likely due to the constraint of the limited number of nucleotides to fill the positions forming the active site. Whether the ribozymes associated with non-LTR retrotransposons and the hepatitis delta virus independently evolved or share a distant common ancestor, the current remarkable similarity of some R2 ribozymes to that of HDV is therefore because these enzymes “converged” on the same sequences. In a similar manner, the constraint on the complexity available for the active site could explain why the termite (Kf) R2 ribozyme (Figure 6A) has the identical active site sequence as an HDV-like ribozyme from a cold tolerant fungus [30].

## Supporting Information

Figure S1 **Protein alignment used to generate the phylogeny of R2.** Sequences comprise the approximately 480 amino acid region carboxy terminal to a conserved AF/YADD motif found in all non-LTR retrotransposable elements.
(PHY)

Figure S2 **Putative ribozyme structures for 18 R2 elements from 13 animal species.** (A) Sequences that comprise regions of the ribozyme as diagrammed in Figure 3A are presented in columns. Nucleotides not base paired in conserved helices are in lower case. Sequences in the P1 column are presented to emphasize R2 elements that cleave in the upstream rRNA (blue text; cleavage site indicated) and those that cleave at the R2 5' end of the element. The sea squirt elements cleave at the 5' ends of the elements, but the 5' ends have sequence similarity to the 28S gene starting at the indicated nucleotide. Species are: flour beetle, *Tribolium* *castaneum* ; gnat, *Rhynchosciaraamericana* ; honeybee, *Apis mellifera*; planaria, *Schmidtea* *mediterranea* ; sea anemone, *Nematostella* *vectensis* ; sea squirt, *Ciona* *intestinalis* ; spider mite, *Tetranychusurticae* ; tadpole shrimp, *Triopscancriformis* ; termite (Kf), *Kalotermes* *flavicollis* ; termite (Rl), *Reticulitermes* *lucifugus* ; tick, *Ixodes* *scapularis* ; zebra finch, *Taeniopygia* *guttata* ; hydra, *Hydra* *magnipapillata*. Multiple lineages of R2 in sea squirt, sea anemone, and flour beetle are differentiated with letters. The ribozyme structures at the 5' ends of the non-LTR elements *Dong* (*B. mori*) and R4 (* Ascaris lumbricoides *) are also presented. Structures that were identical (sea squirt A, tick, zebra finch, R4), had a one nucleotide shift in P1 and P1.1 (tadpole shrimp), or because upstream nucleotides were not included had partial/alternate P1 stems (gnat, both termites) were previously reported [12]. (B) A 5% denaturing acrylamide gel showing the cleavage products for flour beetle A, B, C and *Dong* RNAs in co-transcription/self-cleavage assays. The lower portion of the gel is shown separately to better visualize the 21 nt (7 TAA repeats) *Dong* upstream cleavage product. Assays for earwig, jewel wasp (Nv), and fruit fly RNAs were repeated and included as additional size standards. Uncleaved (red circles) and cleaved products (open red circles) are indicated. Lane M, RNA length markers; fc, fraction cleaved.
(EPS)

Text S1 **Primer sequences used to PCR amplify element (R2, *Dong*) 5' junctions for cloning and analysis in co-transcription/self-cleavage assays.**
(DOC)
