# Supplementary material for: Evolution of the R2 Retrotransposon Ribozyme and Its Self-Cleavage Site
Source: PLoS One. 2013 Sep 16;8(9):e66441. doi: 10.1371/journal.pone.0066441 (PMC3774820; doi:10.1371/journal.pone.0066441)
Supplement: Text S1 — Primer sequences used to PCR amplify element (R2, Dong) 5' junctions for cloning and analysis in co-transcription/self-cleavage assays. (DOC) [file pone.0066441.s003.doc]

Text S1. Primer sequences used to PCR amplify element (R2, Dong) 5’ junctions for cloning and analysis in co-transcription/self cleavage assays.

*Limulus polyphemus*: Lp#3REV, 5'-GAGGTTTTAGTTACAGGCTCG-3'; Lp200REV, 5'-TGGGTTTTAGTGTTCTCCTTC-3'; Lp225REV, 5'-GACATATAGTGGTTGGCGAG-3'; Lp300REV, 5'-TGGGGCCGGTAAGTTCCCATC-3'; Lp28Smut, 5'-TAATACGACTCACTATAGGGAAATTCAACCAAGCGCGGGTAAACGGCCGGAGTAACTATG-3'

*Nasonia vitripennis*: NvB173REV, 5'-CCTTTATTCCGTCGACTGAC-3'; NvB220REV, 5'-GCTGTAAGGCGTCTTCCGATC-3'; NvB310REV, 5'-CCGACCACGCATTGCTTTGTC-3'

*Porcellio scaber*: Ps177REV, 5'-GATAGAAGTTAGCACGGCCTAC-3'; Ps225REV, 5'-GCTTTTTCGAAGCTCTCGG-3'; Ps247REV, 5'-GGGCTGCTGGTGATCTAGC-3'; Ps520REV, 5'-CTTTAATCGCTTAGAGCGATC-3'; PsL3mut, 5'-CGGCGGGAGTAACTATGACTCTCTTTAGATTACTGATAGAAGACTTCCCAGCAGTCCCGC-3'; Ps28S-mut, 5'-TAATACGACTCACTATAGGGAAGCAAGCGCGGGTAAACGGCGGGAGTAACTATGACTCTC-3'

*Anurida maritime*: Am216REV, 5'-GGATTGTTGAACGATCACAATG-3'; Am245REV, 5'-CCCAAGCGCTTTAGCTTTCG-3'; Am300REV, 5'-GATTAAATGTTCCGTGAGGTC-3'

*Bomyx mori* R2: Bm386REV, 5'-GCTGCCCCTCCTCCCGTATC-3'; Bm432REV, 5'-GGGCTGTAGTCCCGCAATGGC-3'; Bm454REV, 5'-CGTTCTAAGGCGGCACT-3'; Bm516REV, 5'-GCACGCCAACAGGGGAGATTAC-3'; Bm590REV, 5'-TTACCACGTGTACAGCCATC-3'; Bm28S_160, 5'-AAGCAAGCGCGGGTAAACGGCGGGAGTAACTATGACTCTCTTACGTCCAACCCTAACGGG-3'

*Bombyx mori* Dong: Dong298REV, 5'-CATCGTGATTTCTGTTGGCGGC-3'; DONG329REV, 5'-GGTTCACATGGGCTATTGCTCC-3'

*Nasonia giraulti*: NgC225REV, 5'-TCTGTGGACCAACGTATCTC-3'; NgC203REV, 5'-GGGCTGGTAACCCATTAG-3'

*Forficula auricularia*: Fa215REV, 5'-CGAGCGAGGAGGCTGGTAAC-3'; Fa300REV, 5'-TCCGAGGCTGGTTTATTGATC-3'; Fa450REV, 5'-TGACACCAGTAAGAGATGGTC-3'

*Samia cynthia*: Sc297REV, 5'-TTGGATTACGCAGCGGCACCAC-3'; Sc303REV, 5'-CTCTTGTTGGATTACGCAGCG-3'; Sc306REV, 5'-CCTCTCTTGTTGGATTACGCAG-3'; Sc310REV, 5'-CCCCCCTCTCTTGTTGGATTAC-3'; Sc315REV, 5'-GAGGTCCCCCCTCTCTTGTTG-3'; Sc361REV, 5'-CGGCGTACACCTCTCGACTAC-3'; Sc389REV, 5'-CGTTCGAAGACCTCCACCTTAGAG-3'; Sc429REV, 5'-CGGGGGAGACAGCGACGGAA-3'; Sc481REV, 5'-CCCAGTGGACAAGCCTGGTCG-3'; ScCatCtoU, 5'-GAGGTCCCCCCTCTCTTGTTGAATTACGCAGC-3'; T7_28S_silk, 5'-TAATACGACTCACTATAGGGTTCAAGCAAGCGCGGGTAAAC-3'; Sc28S_130, 5'-AAGCAAGCGCGGGTAAACGGCGGGAGTAACTATGACTCTCTTCAGTCACGGGTTGCTCAC-3'; Sc28S_165, 5'-AAGCAAGCGCGGGTAAACGGCGGGAGTAACTATGACTCTCTTCCGTCTATGACCGACTAC-3'; Sc28S_235, 5'-AAGCAAGCGCGGGTAAACGGCGGGAGTAACTATGACTCTCTTCCAGATGCGGCGAAGCTC-3'; Sc28S_255, 5'-AAGCAAGCGCGGGTAAACGGCGGGAGTAACTATGACTCTCTTACTCCTACTCTTGACCTG-3'; Sc28S_262, 5'-AAGCAAGCGCGGGTAAACGGCGGGAGTAACTATGACTCTCTTCTCTTGACCTGCACGTGG-3'

*Saturnia pyri*: Sp388REV, 5'-CGGTGGAAACAACGACGGAA-3'; Sp444REV, 5'-ACCCAGGGACAAGCCAGATCG-3'

*Callosamia promethea*: Cp433REV, 5'-CGGGGGAAACAGCGACGGAA-3'; Cp488REV, 5'-CCCAGTGGACAAGCCTGATCG-3'

*Coscinocera hercules*: Ch672REV, 5'-CGGTGGAAACAGCGACGGAA-3'; Ch723REV, 5'-CCCAGTGGACAAGCCTGGTCG-3'

*Tribolium castaneum*: Tc"A"148REV, 5'-CCTACAACTTTCCCCTGCTGAGG-3'; Tc"A"249REV, 5'-TCCTGGTCTAATACGAGGAGGAG-3'; Tc"B"312REV, 5'-GATGCGACGGAACAAACGACTCTTC-3'; Tc"B"366REV, 5'-AGTCTTGTCCGTTAGCCGAACAC-3'; Tc"C"93REV, 5'-CTTTGAAGCCAGTTAAATTTGCCACAG-3'; Tc"C"191REV, 5'-CATAATGATCTTAAATGTCGAAGTGAGATG-3'
